# RELIABILITY OF THE ISOMETRIC DYNAMOMETER IN CONTROL, PARAPLEGIC, AND AMPUTEE INDIVIDUALS

**DOI:** 10.1590/1413-785220233101e255829

**Published:** 2023-02-20

**Authors:** JEFFERSON PACHECO AMARAL FORTES, GISELE HARUMI HOTTA, DÉBORA PINHEIRO AGUIAR, VICTOR BRUNO SOARES DE OLIVEIRA, FRANCISCO CARLOS DE MATTOS BRITO OLIVEIRA, FRANCISCO FLEURY UCHOA SANTOS-JÚNIOR

**Affiliations:** 1 Lead - Dell Research, Development, and Innovation Center, Fortaleza, CE, Brazil.; 2 Instituto Le Santé, Fortaleza, CE, Brazil.; 3 Universidade de São Paulo, Ribeirão Preto Medical School, Department of Health Sciences, Ribeirão Preto, SP, Brazil.; 4 Universidade Estadual do Ceará, Department of Computer Science, Fortaleza, CE, Brazil.

**Keywords:** Data Accuracy, Muscle Strength Dynamometer, Lower Extremity, Confiabilidade dos dados, Dinamômetro de Força Muscular, Extremidade Inferior

## Abstract

**Objective:**

To determine the Intraclass Correlation Coefficient (ICC), Standard Error of Measurement (SEM), Minimum Detectable Change (MDC), and the Minimum Clinically Important Difference (MCID) of the isometric measurements of muscle strength of trunk extension and of flexion and knee extension at maximum contraction in healthy, paraplegic, and amputee individuals, by using an isometric dynamometer with a belt for stabilization.

**Methods:**

An observational cross-sectional study was carried out to assess the reliability of a portable isometric dynamometer in the trunk extension and flexion and knee extension movements of each group.

**Results:**

In all measurements, ICC ranged from 0.66 to 0.99, SEM from 0.11 to 3.73 kgf, and MDC from 0.30 to 10.3 kgf*.* The MCID of the movements ranged from 3.1 to 4.9 kgf in the amputee group and from 2.2 to 3.66 kgf in the paraplegic group.

**Conclusion:**

The manual dynamometer demonstrated good intra-examiner reliability, presenting moderate and excellent ICC results. Thus, this device is a reliable resource to measure muscle strength in amputees and paraplegics. ***Level of Evidence II, Cross-Sectional Study.***

## RESUMO

**Objetivo:**

Determinar o coeficiente de correlação intraclasse (CCI), o erro padrão da medida (EPM), a mínima mudança detectável (MMD) e a mínima mudança clinicamente importante (MMCI) das medidas isométricas de força muscular da extensão de tronco e da flexão e extensão de joelho em contração máxima de indivíduos saudáveis, paraplégicos e amputados, usando um dinamômetro isométrico com cinto para estabilização.

**Métodos:**

Foi realizado um estudo observacional transversal para avaliar a confiabilidade de um dinamômetro portátil isométrico nos movimentos de extensão de tronco e de flexão e extensão de joelho de cada grupo

**Resultados:**

Em todas as medidas o CCI apresentou uma variação de 0,66 a 0,99, o EPM de 0,11 a 3,73 kgf e a MMD de 0,30 a 10,3 kgf. A MMCI dos movimentos variou de 3,1 a 4,9 kgf no grupo de amputados e de 2,2 a 3,66 kgf no grupo de paraplégicos.

**Conclusão:**

O dinamômetro manual demonstrou boa confiabilidade intraexaminador, com variação de CCI de moderada à excelente, apresentando-se como um recurso confiável para mensurar força muscular de amputados e paraplégicos. **Nível de Evidência II, Estudo Observacional Transversal.**

## INTRODUCTION

Global data estimate that about 250,000 to 500,000 individuals suffer some type of spinal cord injury,^1^ caused mostly by traumatic accidents and firearms.^1^ ^),(^ ^2^ Spinal cord injury (SCI) can cause a deficit in strength, with consequent impairment of motor functions, limiting the performance of daily activities^3^ and lowering rates of life expectancy.^4^ Lower limb amputation causes changes in body functions and structures, modifying muscle tone, range of motion, and local sensitivity related to the stump.^5^ Authors estimate that by 2017 about 35.3 million people lived with lower limb amputation due to traumatic accidents.^6^

Individuals with SCI, as well as amputee patients, require attention and monitoring of muscle strength during the rehabilitation process, since sensory-motor deficiencies compromise the functionality of the patient, and these should be identified during clinical evaluation and settled in the intervention process.^7^ ^),(^ ^8^ Several health professionals use muscle strength assessment in their routine, providing quantitative data for criteria of monitoring of functional recovery.^9^ Muscle strength is considered an important physical component for the performance of motor and functional skills; thus, evaluating muscle strength and biomechanical conditions of the patient is relevant for the restoration of daily activities.^10^ ^)-(^ ^12^

Portable dynamometry is one of the methods most used in clinical practice to evaluate muscle strength intensity, because it is easy to handle and low cost, compared to isokinetic dynamometers.^3^ ^),(^ ^13^ ^),(^ ^14^ The muscle manual test is a fast and easily applicable method. Using this tool to evaluate knee flexor and extensor muscles in healthy individuals showed a good reproducibility and it is clinically acceptable when compared with the gold standard of strength measurements, such as the isokinetic dynamometer.^15^

The study that evaluated the intra-examiner reliability of amputees observed the measurements of strength of hip movements, considering the remaining movements depending on the level of amputation.^16^ Changes are expected on the amputated side, such as strength loss and atrophy or hypotrophy; however, the last two can occur not only in the amputated side, but also globally.^17^ Since unilateral amputation leads to changes in gait kinematics, the monitoring of strength gain in the preserved limb is essential in the process of prosthesis adaptation, reinforcing the need to evaluate the remaining limb to plan for the balance recovery, confidence for movement, and gait.^18^

In individuals with paraplegia, upper limbs strength measurements are a way to monitor the treatment evolution of these patients.^18^ The rehabilitation process of paraplegics and amputees should monitor trunk control for sedestation and activation of the lower limbs for assisted orthostatism by using orthotics and gait. However, to our knowledge, no studies evaluate the influence of the condition of rachimedular trauma and unilateral amputation of the lower limbs on muscle strength. Therefore, this study aimed to determine the intra-examiner reliability, measurement errors, and minimal clinically relevant change of an isometric dynamometer in healthy, paraplegic, and amputee individuals.

## MATERIALS AND METHODS

### Study characteristics

A cross-sectional observational study and analysis of measurement properties of an isometric evaluation instrument was performed. The study was approved by the ethics committee of the Hospital Geral de Fortaleza (no. 3,995,609) and all participants read and signed the informed consent form.

### Inclusion and exclusion criteria of the sample

In total, 45 volunteers participated in the study, 15 in the control group, 15 in the group of paraplegics, and 15 in the group of amputees. Adults between 18 and 50 years old, of both sexes, without associated vascular pathologies (coagulation disorders, decompensated diabetes) were eligible for the experiment. Participants of the amputee group should present unilateral transtibial, femoral, or hip amputation and already use a prosthesis for at least six months instead of still being in the adaptation process. In the paraplegic group, participants with bilateral incomplete paraplegia of the lower limbs, without history of pressure ulcers in any part of the body and with stable hemodynamic parameters in the month prior to the study (heart rate, blood pressure, and saturation) were included. In the control group, participants who did not present changes in the lower limbs were included. For all groups, patients’ blood pressure should be stabilized.

Exclusion criteria were individuals who presented associated neuropathological brain alterations such as stroke, Parkinson, Alzheimer, and/or recent traumatic brain injury with cognitive impairment. Individuals with any severe cognitive/psychological dysfunction that could interfere in the performance of the tests, such as panic syndrome, anxiety crises, and/or depression during the evaluation, or individuals with relevant speech impairments that inhibit the communication during tests, were also excluded.

### Dynamometer and use characteristics during tests

**Figure 1:**
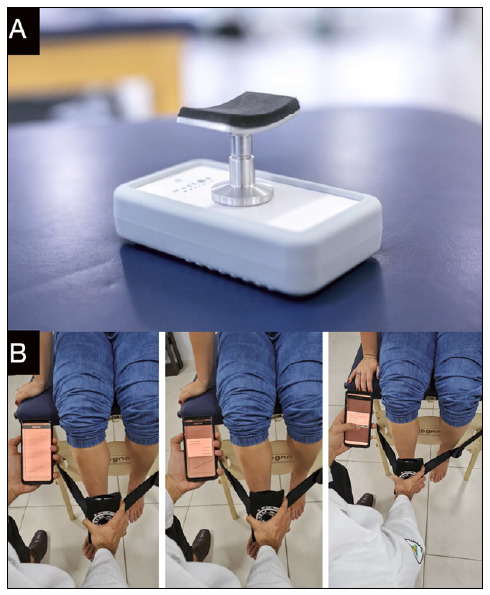
A) Manual dynamometer; B) App operation and reading of information during data collection.

To analyze the isometric strength, the SP Tech^®^ portable isometric dynamometer (Manufactured in Brazil), with the maximum strength capacity of 90.72 kgf (200 lbf), was used. The dynamometer has a Bluetooth function to communicate with the My SP Tech^®^ Android app, installed on a Samsung Galaxy Tab A^®^ tablet, for data collection. By Bluetooth communication, the strength data is sent to the connected device. The Android app shows the strength graph in real time and at the end of the experiment the mean and peak strength values achieved during the experiment are also available to access (Figure 1).

### Evaluator training and familiarization

Initially, the evaluator performed a two-hour training that was applied via internet (online), by a specialized technician of the manufacturer company of the portable dynamometer. During the training, theoretical/practical guidance on how to handle the equipment and app was given and possible questions of the evaluator were clarified. The evaluator, after understanding the equipment operation, performed the pilot test to become acquainted with the movements and ensure that the reading and positioning were adequate. These pilot tests lasted one hour and were carried out for one week, ensuring that the evaluator was able to perform the tests using the device and software.

### Performing strength measures in movements of interest

**Figure 2:**
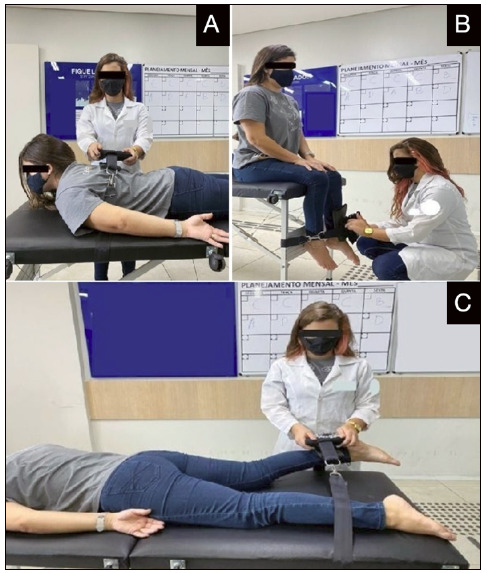
Evaluation with the dynamometer performed in the study. A) Measurement of strength for trunk extension movement; B) Measurement of strength of the knee extensors using the hand dynamometer with the support of a brace; C) Measurement of strength of the knee flexors using the hand dynamometer with the support of a brace.

For each movement, three attempts were performed, with a stimulation of contraction of 15 seconds for each. Between each attempt, a rest period of 15 seconds was given to the participants. During the test, a verbal command was given to the participants at each contraction using the following encouragement expressions: *“*Be strong, be strong, be strong,” “Let’s go” and “To the limit,” The evaluated movements were trunk extension, flexion, and knee extension. For patients with amputations, knee strength was evaluated on the preserved side (Figure 2).

Lumbar spine extensors: Participants remained in ventral decubitus with the upper limb throughout the body. An inelastic stabilization brace was placed around the thorax to position the dynamometer between the lower angles of the scapulae. Then, the patient was asked to elevate the trunk, removing their contact with the stretcher, keeping the cervical in neutral position. The lower limbs were stabilized with another inelastic brace in the medial part of the femur (Figure 1A).^19^ Stabilization was standardized for all patients.

Knee extension: The patient remained seated, with the lower limbs out of the stretcher and their hands relaxed on the legs. The brace was fixed between the stretcher and the lower limbs of the participant and the dynamometer was positioned 15 cm below the anterior tuberosity of the knee. Then, the patient was asked to extend the knee (amputees performed the test with the preserved side) and, during the contraction, there was stabilization of the spine only by voluntary control, allowing the patient to hold the side of the stretcher to avoid compensations (Figure 1B).^20^ This positioning was adopted due to the limitation for posture shifts of patients with paraplegia.

Knee flexion: The patient stayed in ventral decubitus and, by using a belt, the dynamometer was fixed between the sural triceps and the calcaneus tendon (region posterior to the ankle). The participant was asked to bend the knee (amputees performed the test with the preserved side) to measure the strength of the knee flexor muscles (Figure 1C).^21^ The patient was instructed to keep the hip supported on the stretcher to avoid any compensation of movement.

### Statistical analysis

The demographic data of the patients were presented by mean and standard deviation. Data reliability was performed by the mean of the three repetitions during the test and analyzed by the Intraclass Correlation Coefficient (ICC), associated with a 95% confidence interval. These data were interpreted as poor (< 0.40), moderate (≥ 0.40 and ≤ 0.75), and excellent reliability (> 0.75).^22^ The Standard Error of Measurement (SEM) analyzes the error inherent to the instrument associated with a single measurement and was estimated by the formula: SEM=SD×√1-ICC .^23^ The Minimum Detectable Change (MDC) was estimated by the formula: MDC=SEM×1.96×√2 considering a 95% confidence interval.^23^ All analyses were performed using the Statistical Package for the Social Sciences (SPSS, version 24.0; SPSS Inc., Chicago, IL). The estimation of the effect size and the minimum clinically important change (MCIC) was performed according to Armijo-Olivo et al.,^24^ in which were considered moderate effect size equal to or greater than 0.5.^25^

## RESULTS

We evaluated 45 patients. Of the 15 participants in each group, four were from the control group, 12 from the paraplegic group, and nine from the amputee group were males (Table 1). Of those who reported being active and practicing some physical activity, eight individuals were from the control group, 14 from the paraplegic group, and 11 from the amputee group. In the group of amputees, 10 with amputation at the transfemoral level, three of transtibial level, and two at the hip level were included. In the group of paraplegics, the levels of injury were in the thoracic region (T1 to T12).

**Table 1:** Demographic data.

	CONTROL (n = 15)	PARAPLEGIC (n = 15)	AMPUTEES (n = 15)
	Mean (SD)	Mean (SD)	Mean (SD)
Sex (male)	4	12	9
Age (years old)	23 (3)	35 (9)	31 (8)
Height (meters)	1.63 (0.09)	1.65 (0.07)	1.68 (0.11)
Weight (kg)	67.85 (14.72)	73.26 (12.67)	62.13 (14.33)
BMI (kg/m²)	25.51 (4.70)	26.88 (5.12)	21.74 (2.85)
Physically active (n)	8	14	11

The values were expressed as mean ± standard deviation (SD). n: number of participants.

The ICC showed a variation of all measurements from 0.66 to 0.99, presenting a moderate intra-examiner reliability. The percentage of standard error of measurement showed variations from 3% to 50% between all measurements and the minimum detectable change showed variations from 0.30 to 10.3 kgf among all measurements. In the isometric evaluations for trunk extension, the dynamometer presented ICC values from 0.93 to 0.98, demonstrating excellent reliability in all groups evaluated. In the knee flexion movement, the ICC values also demonstrated excellent reliability in all groups, with variations from 0.95 to 0.99. The knee extension movement had ICC values from 0.66 to 0.92, demonstrating a lower value in the control group with moderate reliability; however, the paraplegic and amputee groups presented excellent intra-examiner reliability. The standard error values of the measurement ranged from 1.23 to 2.52 for trunk extension; 0.19 to 3.73 for knee extension; and 0.1 to 1.41 for knee flexion. Tables 2 and 3 show the specific values of each group and each movement.

**Table 2:** Mean and standard deviation (SD) of muscle strength measurements in control, paraplegic, and amputee individuals.

	CONTROL (n = 15)			PARAPLEGIC (n = 15)			AMPUTEE (n = 15)		
	Mean (SD)			Mean (SD)			Mean (SD)		
	TEST 1 (kgf)	TEST 2 (kgf)	TEST 3 (kgf)	TEST 1 (kgf)	TEST 2 (kgf)	TEST 3 (kgf)	TEST 1 (kgf)	TEST 2 (kgf)	TEST 3 (kgf)
Trunk extension	19.01 (9.52)	21.41 (10.13)	22.84 (11.43)	6.88 (4.08)	8.28 (5.25)	8.06 (4.80)	18.76 (10.02)	19.24 (8.89)	19.23 (7.88)
Knee extension	23.47 (6.75)	25.03 (7.37)	24.08 (5.29)	0.38 (0.54)	0.38 (0.65)	0.45 (0.82)	31.95 (11.06)	31.30 (8.98)	32.76 (8.65)
Knee flexion	22.25 (6.18)	21.31 (5.54)	21.28 (5.10)	0.57 (1.03)	0.66 (1.13)	0.63 (1.21)	22.6 (6.37)	23.4 (6.26)	23.15 (6.77)

n: number of participants.

**Table 3:** Intra-evaluator reliability of muscle strength measurements in control, paraplegic, and amputee individuals.

	CONTROL (n = 15)			PARAPLEGIC (n = 15)			AMPUTEE (n = 15)		
	ICC (95%)	SEM (%SEM)	MDC	ICC (95%)	SEM (%SEM)	MDC	ICC (95%)	SEM (%SEM)	MDC
Trunk extension	0.94 (0.85-0.98)	2.52 (0.13)	6.98	0.93 (0.84-0.97)	1.23 (0.18)	3.42	0.98 (0.94-0.99)	1.24 (0.07)	3.44
Knee extension	0.66 (0.18-0.88)	3.73 (0.16)	10.36	0.92 (0.82-0.97)	0.19 (0.50)	0.52	0.87 (0.70-0.95)	3.39 (0.11)	9.41
Knee flexion	0.95 (0.87-0.98)	1.23 (0.05)	3.42	0.99 (0.97-1)	0.11 (0.19)	0.30	0.95 (0.89-0.98)	1.41 (0.06)	3.92

ICC: intraclass correlation coefficient; SEM: standard error of measurement; MDC: minimum detectable change.

The minimum clinically important change in the paraplegic group was 3.66 kgf for trunk extension movement; 2.39 kgf for knee extension movement; and 2.22 kgf for knee flexion movement. The amputee group presented minimum clinically important change values of 4.9 kgf for trunk extension movement; 4.6 kgf for knee extension and 3.1 kgf for knee flexion.

## DISCUSSION

This study determined the intra-examiner reliability of an isometric dynamometer in healthy, paraplegic, and amputee individuals. We observed excellent levels of intra-examiner reliability for trunk extension and knee flexion movements in all groups evaluated. Regarding the knee extension, we found excellent reliability in the paraplegic and amputee groups and moderate reliability in the control group. The performance of muscle strength measurements in amputees^7^ and paraplegics^8^ is a relevant element in the clinical evaluation, as well as the monitoring of the evolution of this population during treatment.

Leijendekkers et al.,^16^ conducted a study with amputees, which one of the objectives was to test the intra-examiner reliability and the validity of the use of portable dynamometer, with and without external stabilization. The result was a good reliability, especially when the techniques were applied with stabilizers during the test. We also used stabilization devices in our study, obtaining a satisfactory intra-examiner reliability in all movements evaluated. Another study in the literature observed that the evaluation performance of muscle strength of the lower limbs of physically active individuals tends to present difficulties in the necessary stabilization, because it depends on the strength that the examiner should have to avoid compensation of other joints.^26^ The brace use minimizes the stabilization difficulties, which may favor the results of the evaluation and provide greater reliability.^9^ We evaluated the reliability of the movements of the present study by using non-elastic straps, which may have allowed greater stabilization of movements and minimized the evaluator influence, contributing to better values of intra-examiner reliability.

Trunk control is crucial for postural stability and propulsion between^27^ paraplegics and amputees and their muscle strength should be a widely investigated element throughout the therapeutic process. The present study showed that the amputation in one of the lower limbs did not affect the ability to generate enough muscle strength for trunk extension to impact reliability values. Moreover, the use of isometric dynamometers may be an alternative to other low-reliability assessment tools, such as manual tests,^28^ offering a greater accuracy regarding the clinical evolution of the evaluated individuals.

### Strengths and limitations of the study

This is a pioneering study that analyzed the intra-examiner reliability of an isometric dynamometer, to the best our knowledge, not yet scientifically evaluated, and in clinical conditions little investigated such as amputation and paraplegia. The study presents limitations regarding the sample size, according to the guidelines of COSMIN for conducting reliability studies, and presents methodological limitations due to the absence of the inter-examiner reliability measure. We emphasize that the limitations mentioned are mainly related to the difficulties of this population in the displacement to the research center due to the social distancing resulting from the COVID-19 pandemic. Moreover, we included as a limitation the non-stabilization of the hip in the evaluation of knee flexors. We emphasize that the reproducibility of our data depends on the individual’s positioning and stabilization with inelastic straps according to the methodology proposed in this study.

## CONCLUSION

We identified that the intra-examiner reliability of the equipment used varied from moderate to excellent in the control group and was excellent in the amputee and paraplegic groups for the analyzed movements.
